# Effect of High-Entropy Spinel Ferrite (Mn_0.2_Zr_0.2_Cu_0.2_Ca_0.2_Ni_0.2_)Fe_2_O_4_ Doping Concentration on the Ferroelectric Properties of PVDF-Based Polymers

**DOI:** 10.3390/polym15122688

**Published:** 2023-06-15

**Authors:** Jiale Qiao, Zhaoting Liu, Haiwei Mu, Chao Liu

**Affiliations:** 1 School of Physics and Electronic Engineering, Northeast Petroleum University, No. 199, Fazhan Road, Daqing 163318, China; qiaojiale@stu.nepu.edu.cn (J.Q.); liuchao@nepu.edu.cn (C.L.); 2 School of Electrical Engineering, Suihua University, Suihua 152001, China; 3 School of Physics and Electronic Engineering, Northeast Petroleum University Qinhuangdao, No. 550, West Hebei Street, Qinhuangdao 066004, China

**Keywords:** high-entropy spinel ferrite, polymer, dielectric, ferroelectric, energy density

## Abstract

Polyvinylidene fluoride (PVDF)-based dielectric energy storage materials have the advantages of environmental friendliness, high power density, high operating voltage, flexibility, and being light weight, and have enormous research value in the energy, aerospace, environmental protection, and medical fields. To investigate the magnetic field and the effect of high-entropy spinel ferrite (Mn_0.2_Zr_0.2_Cu_0.2_Ca_0.2_Ni_0.2_)Fe_2_O_4_ nanofibers (NFs) on the structural, dielectric, and energy storage properties of PVDF-based polymers, (Mn_0.2_Zr_0.2_Cu_0.2_Ca_0.2_Ni_0.2_)Fe_2_O_4_ NFs were prepared via the use of electrostatic spinning methods, and (Mn_0.2_Zr_0.2_Cu_0.2_Ca_0.2_Ni_0.2_)Fe_2_O_4_/PVDF composite films were prepared via the use of the coating method. The effects of a 0.8 T parallel magnetic field, induced for 3 min, and the content of high-entropy spinel ferrite on the relevant electrical properties of the composite films are discussed. The experimental results show that, structurally, the magnetic field treatment causes the originally agglomerated nanofibers in the PVDF polymer matrix to form a linear fiber chain with different fiber chains parallel to each other along the magnetic field direction. Electrically, the introduction of the magnetic field enhanced the interfacial polarization, and the (Mn_0.2_Zr_0.2_Cu_0.2_Ca_0.2_Ni_0.2_)Fe_2_O_4_/PVDF composite film with a doping concentration of 10 vol% had a maximum dielectric constant of 13.9, as well as a low energy loss of 0.068. The high-entropy spinel ferrite (Mn_0.2_Zr_0.2_Cu_0.2_Ca_0.2_Ni_0.2_)Fe_2_O_4_ NFs and the magnetic field influenced the phase composition of the PVDF-based polymer. The α-phase and γ-phase of the cohybrid-phase B1 vol% composite films had a maximum discharge energy density of 4.85 J/cm^3^ and a charge/discharge efficiency of 43%.

## 1. Introduction

The dielectric material is the main component of a dielectric capacitor. Its bound charge can be shifted in a microscopic range under an external electric field and then polarized, allowing electrostatic energy to be stored in the displaced dipole, corresponding to the charging process of a dielectric capacitor; when the polarized capacitor is connected to an external conductive path, the polarized dipole is depolarized, corresponding to the discharge process of a dielectric capacitor [1,2]. A typical dielectric capacitor is a simple sandwich structure in which only metal electrodes are plated on both sides of the surface of the dielectric material. Compared with common energy storage devices, such as lithium-ion batteries, supercapacitors, and electrochemical storage, dielectric capacitors have the advantages of a high power density, light mass, easy preparation, low price, and high operating voltage. They are widely used in new energy vehicles, flexible wearable electronic devices, power inverters, aerospace, pulsed military weapons, electronic medical devices, and other fields [3,4,5,6].

An important parameter for measuring the performance of a dielectric capacitor is the discharge energy density, which can be calculated via Equation (1):

(1) $$Udischarge=\int_{Dmin}^{Dmax}EdD$$

where *U_discharge_*, *E*, and *D* represent discharge the energy density, electric field, and electric displacement, respectively. *D* and polarization (*P*) are related to $D=\varepsilon_{0}\varepsilon_{r}E=\varepsilon_{0}$*E* + *P*; the value of $\varepsilon_{r}$ is the relative dielectric constant pertaining to the material’s own properties, and $\varepsilon_{0}$ is the vacuum dielectric constant. For linear dielectrics, the discharge energy density can also be expressed as Equation (2):

(2) $$Udischarge=\frac{1}{2}\varepsilon_{0}\varepsilon_{r}E_{b}^{2}$$

High discharge energy density often requires large *E_b_* and $\varepsilon_{r}$; *E_b_* and $\varepsilon_{r}$ are mainly related to the a dielectric material’s own performance, so the performance of a dielectric material determines the energy storage of a dielectric capacitor.

Common dielectric materials include ceramics and polymer materials. Ceramic materials include CaCu_3_Ti_4_O_12_ [7,8], BaTiO_3_ [9,10,11], TiO_2_ [12,13,14], SrTiO_3_ [15,16,17], and other ABO_3_ perovskites [18].

Polymer materials can be divided into linear dielectric materials and nonlinear dielectric materials, according to whether the dielectric constant varies linearly or nonlinearly with frequency. Linear dielectric materials include PMMA, PI, PET, etc. [19,20]. Nonlinear dielectric materials include PVDF and copolymers. Among the polymer materials, PVDF has a relatively large dielectric constant and energy density [21,22], much greater than the dielectric *K* (~2.2) and discharge energy density (~4 J/cm^3^) of commercial BOPP [23,24], which is an ideal candidate for the innovative design and miniaturization of future power as well as electronic equipment [25].

PVDF is a semicrystalline polymer, generally consisting of 59.4% fluorine and 3% hydrogen [26]. PVDF is more chemically stable than polymers composed of H-C bonds due to the greater negative charge of the F atom and the high dissociation energy of the C-F bond. Commercial PVDF is polymerized in suspensions or emulsions via the use of free radical initiators to form -CH_2_-CF_2_ repeating units. -CH_2_-CF_2_ is arranged in different spatial arrangements along the polymer chain to produce different phases. The different phase contents are influenced by the polymerization step and method, molecular weight and distribution, thermal history, and cooling rate, which can affect the properties of PVDF [27]. PVDF contains at least four crystalline phases: the α-phase is a TGTG trans conformational structure with H and F atoms alternately dispersed on both sides of the polymer chain [28]; the β-phase is a TTTT fully planar sawtooth structure; the γ-phase is a TTTG triple trans structure with the dipole orientation towards the chain axis; and the δ-phase is a polar version of the α-phase. Of these, the polarity of the α-phase is the most stable and is less easily polarized, to the detriment of ferroelectric properties. The polar β-phase produces a net dipole moment almost perpendicular to the polymer chains, pointing from electronegative fluorine to positively charged hydrogen, and these chains can crystallize into a quasi-hexagonal, tightly packed β-phase structure in which the dipoles of all chains are aligned, with better piezoelectric effects, pyroelectric effects, and higher initial polarization, but with a large residual polarization affecting energy storage [29,30]. Many studies have shown that the polar γ-phase can be seen as an intermediate between the α-phase and β-phase, with ferroelectric effects favoring energy storage [31,32,33,34].

Various studies have shown that polymeric materials are flexible, lightweight, and have a high breakdown voltage but low dielectric constant. Ceramic materials have higher dielectric constants but tend to have lower breakdown strengths, complementing the electrical properties of polymer materials [35]. To improve the electrical properties of polymers, ceramic materials with high dielectric constants are added to polymer matrices to form ceramic/polymer composites [36,37,38,39,40,41,42].

The large number of means of preparing composite nanomaterials demonstrate the desire for the cocktail effect, with abundant attempts to enhance the energy storage properties of polymeric dielectric materials; however, recent experiments in many fields have shown that high-entropy materials, with their inherent advantages of the cocktail effect, phase stability, lattice distortion of atomic disorder, and slow diffusion kinetics have come under the spotlight in recent years for the development of new materials [43,44,45]. Some high-entropy materials have been shown to have excellent dielectric properties [46,47]. Therefore, the use of high-entropy materials to enhance the electrical properties of PVDF is an important experimental program.

The definition of entropy, in thermodynamics, and the phase stability of high-entropy carbides (HECs) are determined by Gibbs free energy, which can be defined by Equation (3) [48]:

(3) $$\Delta G_{mix}=\Delta H_{mix}-T\Delta S_{mix}$$

where *H_mix_* is the enthalpy of mixing and *S_mix_* is the entropy of mixing, which can be expressed as Equation (4):

(4) $$\Delta S_{conf}=-R\sum_{x_{i}}(x_{i}\sum_{n}c_{j}\ln c_{j})$$

where *R*, *x_i_*, *n*, and *c_j_* are the gas constant, fraction of the sublattice, *i*, number of components, and atomic fraction of components, *j*, respectively. Larger entropy favors lower Gibbs free energy and, hence, phase stability.

Great progress has been made in the field of dielectric capacitors. The main means of improving the dielectric and energy storage properties of polymers are multilayered, but the preparation process is complex and not suitable for commercialization. Single-layer films doped with conductive substances, such as metal ions and carbon nanotubes [49,50], have been studied for insulating materials such as doped ceramics [36,37,38,39,40,41,42], but there is less literature on semiconductor high-entropy materials as a filler phase with which to modify PVDF-based semicrystalline polymers. Some literature has shown that spinel ferrite can improve dielectric and breakdown properties [51,52].

The aim of this study is to use semiconducting high-entropy spinel ferrite (Mn_0.2_Zr_0.2_Cu_0.2_Ca_0.2_Ni_0.2_)Fe_2_O_4_ NFs as a filler phase with which to modify semicrystalline polymer PVDF. A noteworthy problem, however, is that the agglomeration of high-entropy spinel ferrite in the PVDF base was detrimental to interfacial compatibility and breakdown. The corresponding solution is to apply a magnetic field to the surface of the parallel film and use the magnetic field force to deagglomerate the agglomerated high-entropy spinel ferrite; some evidence suggests that the interaction of the magnetic field with the magnetic particles produces a magnetoelectric effect that optimizes the dielectric properties. In summary, the effect of the applied magnetic field on the crystal structure, dielectric, and energy storage properties of (Mn_0.2_Zr_0.2_Cu_0.2_Ca_0.2_Ni_0.2_)Fe_2_O_4_/PVDF has been investigated.

## 2. Materials and Methods

### 2.1. Materials

Polyvinylidene fluoride (PVDF) was purchased from Shanghai Sanfu New Materials Co., Ltd., China. Polyvinyl pyrrolidone and iron nitrate (Fe(NO_3_)_2_•9H_2_O) were purchased from Shanghai MACKLIN Biochemical Technology Co. Anhydrous ethanol (C_2_H_6_O), copper nitrate (Cu(NO_3_)_2_•3H_2_O), calcium nitrate (Ca(NO_3_)_2_•4H_2_O), nickel nitrate (Ni(NO_3_)_2_•6H_2_O), and N,N-dimethylformamide (DMF) were purchased from Shanghai Sinopharm Group Chemical Reagent Co. Zirconium nitrate (Zr(NO_3_)_4_•5H_2_O), manganese nitrate (Mn(NO_3_)_2_•4H_2_O), and polyvinyl pyrrolidone were purchased from Shanghai Aladdin Biochemical Technology Co. (Shanghai, China). Citric acid was purchased from Shanghai Sinopharm Chemical Reagent Co., Ltd., China. Deionized water was self-made, and all of the chemicals as well as reagents were analytically pure and not further processed.

### 2.2. Synthesis of High-Entropy Spinel Ferrite (Mn_0.2_Zr_0.2_Cu_0.2_Ca_0.2_Ni_0.2_)Fe_2_O_4_ NFs

**Figure 1:**
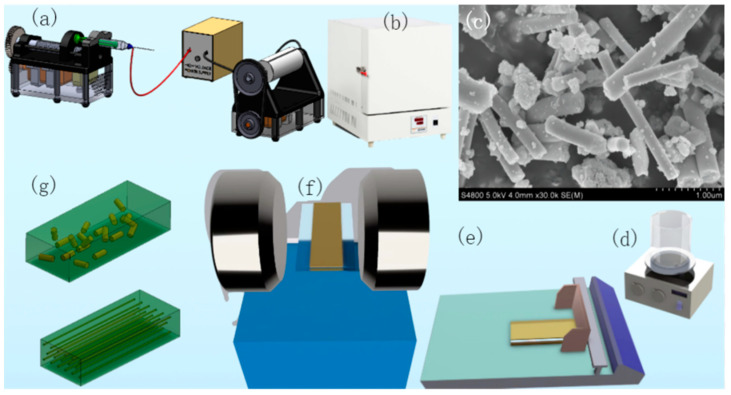
(**a**) Preparation of high-entropy spinel ferrite (Mn_0.2_Zr_0.2_Cu_0.2_Ca_0.2_Ni_0.2_)Fe_2_O_4_ NFs via electrostatic spinning, (**b**) calcined (Mn_0.2_Zr_0.2_Cu_0.2_Ca_0.2_Ni_0.2_)Fe_2_O_4_ NFs, and (**c**) fiber morphology after calcination (**d**–**g**).

High-entropy spinel ferrite (Mn_0.2_Zr_0.2_Cu_0.2_Ca_0.2_Ni_0.2_)Fe_2_O_4_ NFs were prepared via the sol–gel method and electrostatic spinning method. The main preparation steps are shown in Figure 1. Firstly, 0.2 mol of Fe(NO_3_)_2_•9H_2_O was weighed; hydrated nitric acid composed of 0.02 mol of Cu(NO_3_)_2_•3H_2_O, Ca(NO_3_)_2_•4H_2_O, Ni(NO_3_)_2_•6H_2_O, Zr(NO_3_)_4_•5H_2_O, and Mn(NO_3_)_2_•4H_2_O, 2.5 g of citric acid C_6_H_8_O_7_, and 2 g of polyvinyl pyrrolidone were then dissolved in 6 mL of deionized water and 20 mL of anhydrous ethanol. The mixture was stirred vigorously to obtain solution A. After the complete reaction, the solution was stirred for 6 h to obtain solution B. Solution B was then spun using an electrostatic spinning machine, as shown in Figure 1a. The distance between the needle and the receiver was adjusted to 15 cm, and the working voltage was set to 20 kV. After half an hour, the amorphous mixed fibers obtained from the roller receiver were calcined in a muffle furnace at 600 °C under air conditions for 3 h, as shown in Figure 1b. High-entropy spinel ferrite was obtained. SEM images are shown in Figure 1c.

### 2.3. Fabrication of (Mn_0.2_Zr_0.2_Cu_0.2_Ca_0.2_Ni_0.2_)Fe_2_O_4_/PVDF Nanocomposite Film

As shown in Figure 1d, five 50 mL beakers were filled with 30 mL of DMF, PVDF powder, and (Mn_0.2_Zr_0.2_Cu_0.2_Ca_0.2_Ni_0.2_)Fe_2_O_4_ NFs doped at 1 vol%, 3 vol%, 5 vol%, 7 vol%, and 10 vol%, respectively; the mixture in each beaker was stirred for 12 h to obtain solutions A, B, C, D, and E, respectively. As shown in Figure 1e, each solution was slowly poured onto 2 clean glass plates and scraped uniformly through the use of a coating machine to form a uniform composite film. A scraped film was placed in a drying oven at 180 °C for 10 min and then removed as well as quenched in a mixture of deionized water and ice water, cooled down, and then placed in a drying oven at 80 °C for 6 h. The other composite film was placed in a uniform parallel magnetic field at 0.8 T for 3 min immediately after scraping, as shown in Figure 1f, and then placed in a drying oven at 180°; the procedure was repeated for the first glass plate. Figure 1g shows a model of the distribution of high-entropy spinel ferrite within the composite film before and after magnetic field treatment.

### 2.4. Characterization

The crystal structure information of high-entropy spinel ferrite (Mn_0.2_Zr_0.2_Cu_0.2_Ca_0.2_Ni_0.2_)Fe_2_O_4_ with composite (Mn_0.2_Zr_0.2_Cu_0.2_Ca_0.2_Ni_0.2_)Fe_2_O_4_/PVDF films was observed through was carried out via X-ray diffraction (Bruker D8 Advance) with an angle range of 10–90°. Elemental analysis was carried out with an X-ray fluorescence spectrometer (XRF), a ZSX Primus model, Rigaku, Japan. A field emission SEM (ZEISS Merlin Compact, Jena, Germany) was used to collect the surface micromorphological information of the composite films, and the micromorphological information of the semiconductor high-entropy spinel ferrite nanofibers was observed via the use of a Japanese S4800 Nikon scanning electron microscope. For a clear view of the fiber surface morphology and to prevent the agglomeration of the powder, it was placed in an ultrasound machine at 100 w power for 3 min before nanofiber SEM testing. The functional groups of the composite film materials were characterized via Fourier-transform infrared spectroscopy (FTIR). The TG and DSC were tested with a synchronous thermal analysis tester (NETZSCH STA 449F3) produced by NETZSCH Company in Selb, Germany. The test temperatures were 100–600 °C and 60–200 °C, and the heating rate was 10.0 K/min.

Prior to the electrical performance tests, the composite films were coated with Ag metal electrodes on both sides in order to form a typical sandwich-structured dielectric capacitor. The dielectric properties of the composite films were measured via the use of a model Alpha-A broadband dielectric spectrometer from Novocontrol, Germany, in the frequency range of 1 Hz to 10^7^ Hz. The ferroelectric properties were measured via the use of a Radiant Premier II ferroelectric test system at a frequency of 10 Hz. The discharge energy density and charge/discharge efficiency were calculated from the hysteresis lines.

## 3. Results and Discussion

Figure 1c shows an SEM of the powder, with large-aspect-ratio nanofibers with end diameters of approximately 200–400 nm and lengths of 1–2 μm. A small number of particles were present in the prepared fibers, possibly due to shredding from fiber breakage caused by excessive ultrasonic power or time during the SEM pretreatment process. Alternatively, the needle was too fine during the electrostatic spinning process, the spinning fluid was viscous, and the needle was subjected to high thrust, resulting in the occasional agglomeration of large Taylor cone jets during the spinning process, resulting in agglomeration.

**Figure 2:**
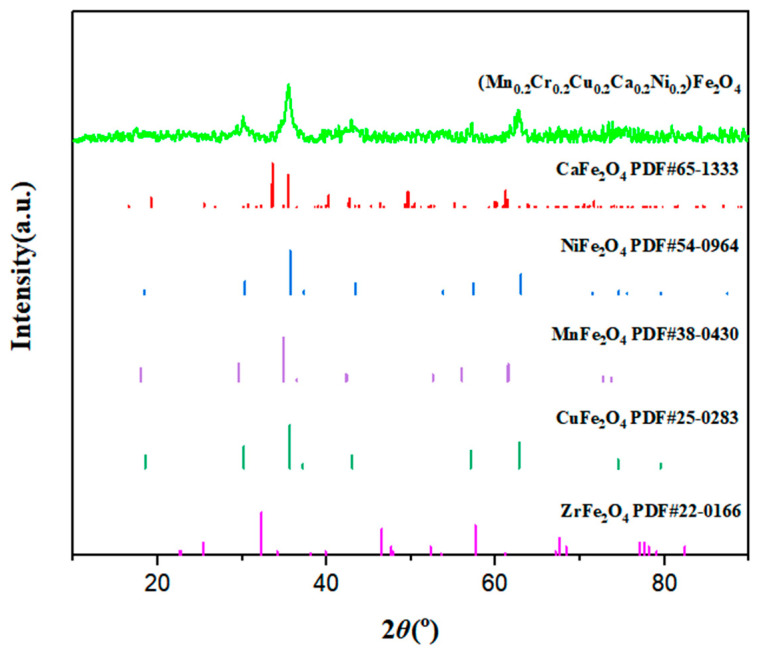
XRD of (Mn_0.2_Zr_0.2_Cu_0.2_Ca_0.2_Ni_0.2_)Fe_2_O_4_ NFs.

Figure 2 shows the X-ray diffraction pattern of (Mn_0.2_Zr_0.2_Cu_0.2_Ca_0.2_Ni_0.2_)Fe_2_O_4_ NFs and the simplified characteristic peaks of a standard PDF card of spinel ferrite containing the elements. The (Mn_0.2_Zr_0.2_Cu_0.2_Ca_0.2_Ni_0.2_)Fe_2_O_4_ NFs have five characteristic peaks: 30.1°, corresponding to the (220) crystal plane of NiFe_2_O_4_ and CuFe_2_O_4_; 35.5°, the main peak corresponding to the (311) crystal plane of CaFe_2_O_4_, NiFe_2_O_4_, and CuFe_2_O_4_; 43°, corresponding to the (311) crystal plane of CuFe_2_O_4_ and CaFe_2_O_4_; 57°, corresponding to the (511) crystal plane of NiFe_2_O_4_ and ZrFe_2_O_4_; and 63°, corresponding to the (440) crystal plane of CuFe_2_O_4_. It was demonstrated that the high-entropy spinel ferrite contains several elements of Fe, Mn, Zr, Cu, Ca, and Ni.

The quantitative composition of (Mn_0.2_Zr_0.2_Cu_0.2_Ca_0.2_Ni_0.2_)Fe_2_O_4_ NFs was tested via XRF, as shown in Table 1. The contents of Mn, Zr, Cu, Ca, Ni, and Fe in the corresponding oxides were 4.64%, 8.4%, 5.16%, 3.43%, 4.53%, and 45.51%, respectively. Each mol of (Mn_0.2_Zr_0.2_Cu_0.2_Ca_0.2_Ni_0.2_)Fe_2_O_4_ contains Mn, Zr, Cu, Ca, Ni, and Fe in a ratio of about 0.2:0.2:0.2:0.2:0.2:2. Combining XRD, SEM, EDS, and XRF, high-entropy spinel ferrite (Mn_0.2_Zr_0.2_Cu_0.2_Ca_0.2_Ni_0.2_)Fe_2_O_4_ NFs were prepared.

**Figure 3:**
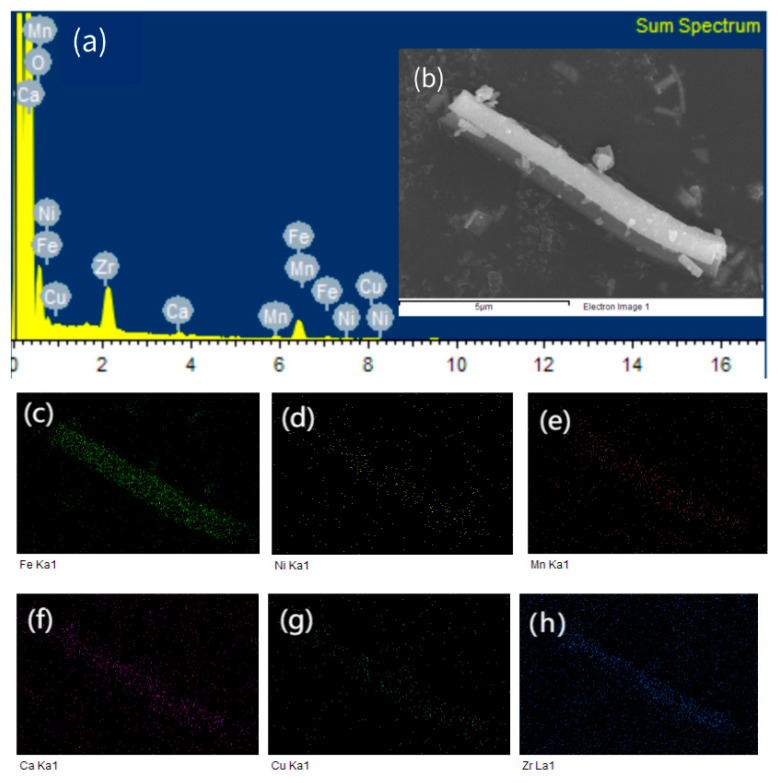
(**a**) EDS of (Mn_0.2_Zr_0.2_Cu_0.2_Ca_0.2_Ni_0.2_)Fe_2_O_4_ NFs; (**b**) SEM of (Mn_0.2_Zr_0.2_Cu_0.2_Ca_0.2_Ni_0.2_)Fe_2_O_4_ NFs; (**c**–**h**) mapping of Fe, Ni, Mn, Ca, Cu and Zr respectively.

Figure 3a shows the fiber EDS energy spectrum, which shows the composition of the target elements, and Figure 3b shows the microscopic morphology of a single fiber, which can be seen in Figure 3c–h as mapping high-entropy spinel ferrite containing the elements Fe, Ni, Mn, Ca, Cu, and Zr, respectively. It can be seen that the elements are uniformly distributed on the fiber’s surface.

**Figure 4:**
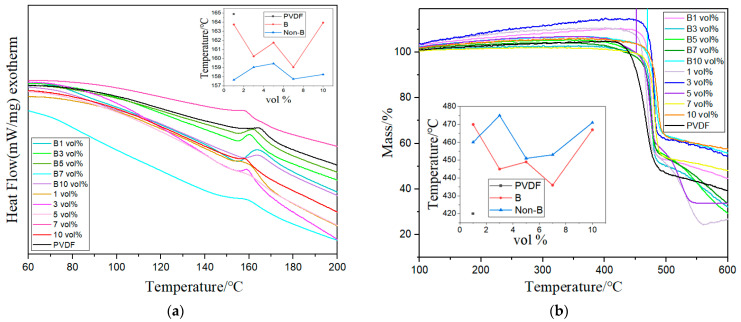
(**a**,**b**) DSC and TG of the composite film.

The melting peak temperature of pure PVDF shown in Figure 4a is 165 °C, which is reduced by doping (Mn_0.2_Zr_0.2_Cu_0.2_Ca_0.2_Ni_0.2_)Fe_2_O_4_ NFs. The melting temperature of magnetic-field-treated composite films is generally higher than that of non-magnetic-field-treated composite films. The crystallinity of the magnetic-field-treated composite film is also higher, with B3 vol% having the highest crystallinity.

Figure 4b shows the thermal weight loss curve of PVDF-based composite films. Pure PVDF decomposes at 420 °C, and the decomposition temperature is greatly increased through doping (Mn_0.2_Zr_0.2_Cu_0.2_Ca_0.2_Ni_0.2_)Fe_2_O_4_ NFs. When the doping concentration is 3 vol% the maximum decomposition temperature is 476 °C, indicating that (Mn_0.2_Zr_0.2_Cu_0.2_Ca_0.2_Ni_0.2_)Fe_2_O_4_ NFs improve the thermal stability of the composite films. The external magnetic field slightly reduced the decomposition temperature, but still exceeded the thermal decomposition temperature of pure PVDF.

**Figure 5:**
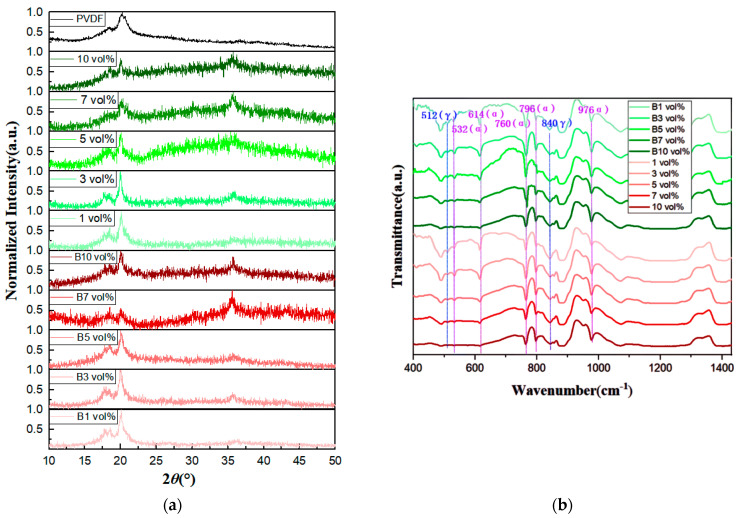
(**a**,**b**) XRD and FTIR of the composite film.

The diffraction peaks of the composite films were observed through XRD diffraction patterns, as shown in Figure 5a. The 1 vol% diffraction peaks of the film without magnetic field treatment were 18.45° and 20.02°, corresponding to the (020) and (110) crystallographic planes, respectively, of the γ-phase of PVDF. The 3 vol% diffraction peaks were 17.71°, 18.38°, and 19.90°, corresponding to the (100), (020), and (110) crystallographic planes, respectively, of the α-phase of PVDF. The 5 vol% diffraction peaks were 17.94°, 18.32°, and 20.06°, corresponding to the (110), (020), and (110) crystallographic planes, respectively, of the α-phase of PVDF. The 7 vol% diffraction peaks were 20.01° for the (110) crystal faces of the γ-phase of PVDF. The 10 vol% diffraction peaks were 18.57° and 19.96° for the (020) and (110) crystal faces, respectively, of the α-phase of PVDF [53].

The diffraction peaks of the magnetic-field-treated films’ B1 vol% were 17.89°, 18.57°, and 20.08°, corresponding to the (100) as well as (020) crystal planes of the α-phase and the (110) crystal plane of the γ-phase, respectively. The diffraction peaks of B3 vol% were 17.79°, 18.38°, 19.96°, and 20.2°, corresponding to the (100), (020), and (110) crystal planes of the α-phase in addition to the (110) crystal plane of the γ-phase, respectively. The diffraction peaks of B5 vol% were 18.51° and 20.1°, corresponding to the (020) crystal plane of the α-phase and the (110) crystal plane of the γ-phase, respectively. The diffraction peaks of B7 vol% were 17.84°, 18.47°, 20.02°, and 20.3°, corresponding to the (100), (020), and (110) crystal faces, respectively, of the γ-phase. The diffraction peaks of B10 vol% were 17.89°, 18.47°, 20.02°, and 20.3°, corresponding to the (100), (020), and (110) crystal faces, respectively, of the γ-phase. The characteristic peaks of high-entropy spinel ferrite at low doping concentrations are not obvious in the above films. At doping concentrations greater than or equal to 3 vol%, the diffraction peaks of 35.5° high-entropy spinel ferrite start to appear. The characteristic peaks are particularly pronounced at doping concentrations greater than or equal to 7 vol%. The doping of the magnetic field and high-entropy spinel ferrite induces the development of the γ-phase, and in some of the composite films this coexists with the most stable α-phase.

In Figure 5b, the FTIR spectrum shows that the effect of the magnetic field is less than the effect of the filler on the phases. As the doping concentration of (Mn_0.2_Zr_0.2_Cu_0.2_Ca_0.2_Ni_0.2_)Fe_2_O_4_ NFs increases, the γ-phase peaks at 512 cm^−1^ and 840 cm^−1^ wavelengths are evident, and the nonpolar γ-phase in the composite films can be observed near 440 cm^−1^ and 431 cm^−1^ at a doping concentration of 10 vol%; the nonpolar α-phase can be retrieved near 532 cm^−1^, 614 cm^−1^, 760 cm^−1^, 796 cm^−1^, and 976 cm^−1^ [54]. The two main conformations of the ferroelectric polymer in the all-organic composite media are the α-phase and γ-phase, which are consistent with the characterization results of the XRD pattern.

**Figure 6:**
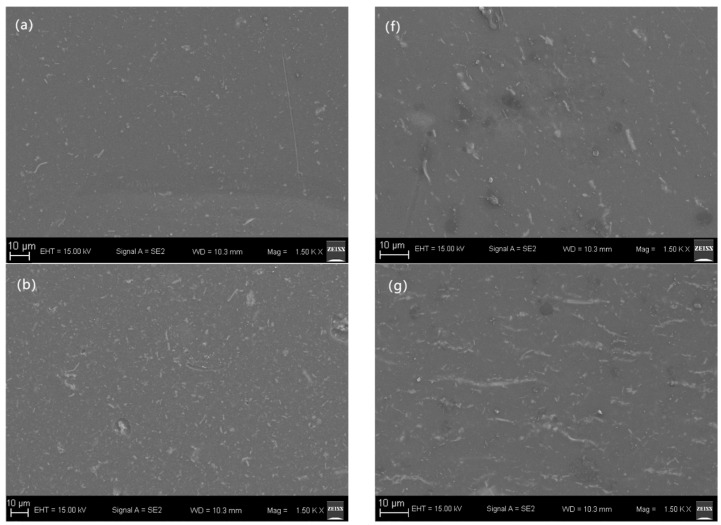

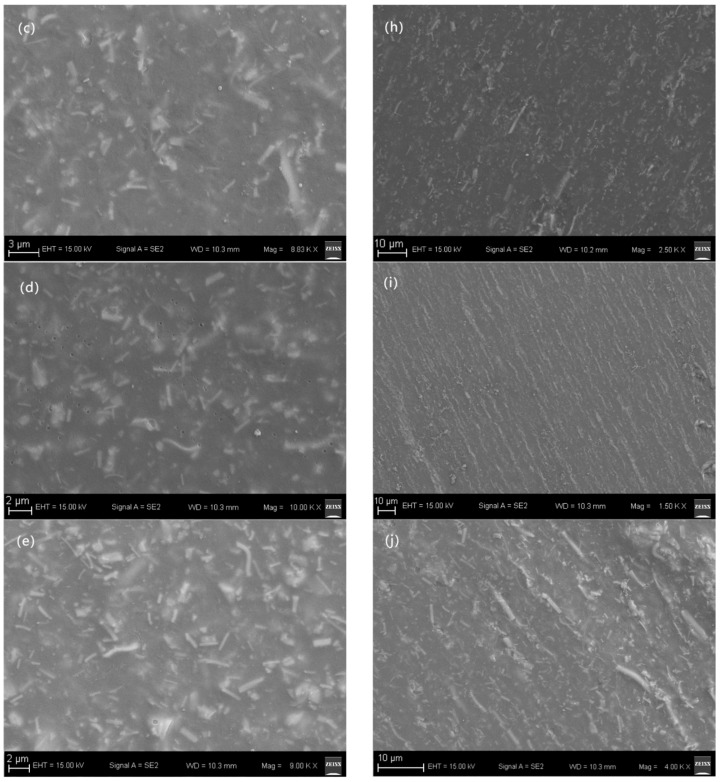
SEM of the composite film.

Figure 6 shows the distribution state of high-entropy spinel ferrite on the surface of the composite film, where Figure 6a–e corresponds to the SEM of 1 vol%, 3 vol%, 5 vol%, 7 vol%, and 10 vol%, respectively. Figure 6b–j corresponds to the SEM of B1 vol%, B3 vol%, B5 vol%, B7 vol%, and B10 vol%, respectively. The surface of the composite films (Figure 6a–e) without magnetic field treatment shows that the fibers are uniformly dispersed on the surface of the composite films, with the fiber ends randomly oriented in different directions, and shows that the fiber density gradually increases with increasing doping concentration. The magnetic-field-treated composite films (Figure 6f–j) show a change in the orientation of the fibers under the action of the magnetic field force, with the originally disordered fibers aligned in the direction of the magnetic field, forming parallel lines between them, which gradually increase as the doping concentration increases, with similar parallel lines joining end-to-end to form longer chains up to tens of microns long.

**Figure 7:**
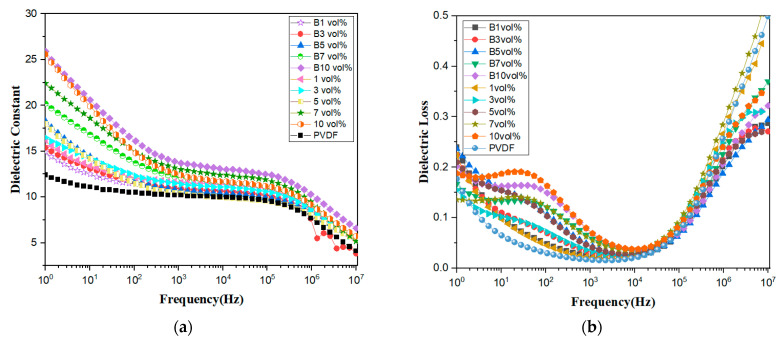
(**a**,**b**) Dielectric constants and dielectric losses of the composite film.

It is very important to study the effect of doping concentration and applied parallel magnetic field on the dielectric properties of composite materials. In Figure 7a, the dielectric constant curve shows that, whether or not it is treated with a magnetic field, the dielectric constant increases at lower frequencies as the doping concentration increases. The dielectric constant of low-concentration-doped composite films does not change much before and after magnetic field treatment, due to the low doping concentration, the increased distance between fibers, and because the fibers do not interact with each other. The applied magnetic field only changes the orientation of the fibers and does not affect the interfacial compatibility of fibers with the PVDF base. Until the doping concentration reached 10 vol%, the dielectric constant of magnetic-field-treated composite films at 1 kHz was significantly greater than that of composite films without the effect of a magnetic field, at 13.9 and 12.6, respectively. The magnetic field had an optimizing effect on the dielectric constant for the high concentration of doping. Pure PVDF has the smallest dielectric constant, 11, at 1 kHz.

Figure 7b shows the loss values for the 10 composite films, all of which were less than 0.1 at 1 kHz. At low frequencies (<10^4^ Hz) the curve rises, indicating that, as the doping concentration increases, it is equivalent to the introduction of a large number of impurities in the polymer, which together with the pores within the composite leads to an increase in DC conductivity loss, at which point the dipole polarization loss is negligible; the higher the doping concentration, the greater the loss. As the frequency increases (>10^4^ Hz), dipole polarization gradually dominates and the dipole flip rate is less than the increase in frequency, causing internal friction between the electric dipoles, which in turn increases the dielectric loss. The composite films with low-concentration doping of 1 vol% demonstrated the smallest loss, which increased slightly with an increase in the doping concentration. The losses for the same doping concentration in the ten samples almost overlapped two by two, indicating that the magnetic field has little effect on the loss. B10 vol% had the largest dielectric constant, 13.9 at 1 kHz, and B1 vol% had a dielectric loss of 0.025. On balance, the sample with the best dielectric properties was B10 vol%, with a dielectric constant and loss of 13.9 and 0.068, respectively.

Breakdown is related to the inherent band gap width of the material itself in addition to external factors, such as mechanical deformation and thermal breakdown. The breakdown strength is calculated via the use of the two-parameter Weibull distribution of Equation (5):

(5) $$P(E)=1-\exp{\left(\frac{E}{E_{b}}\right)}^{\beta }$$

where *P*(*E*) represents the cumulative probability of electrical failure, *E* is the experimental electric field strength at the time of electrical breakdown, *E_b_* is the characteristic breakdown strength at a cumulative failure probability of 63.7%, and *β* is the shape parameter used to evaluate the degree of breakdown strength dispersion, with larger values indicating higher accuracy.

**Figure 8:**
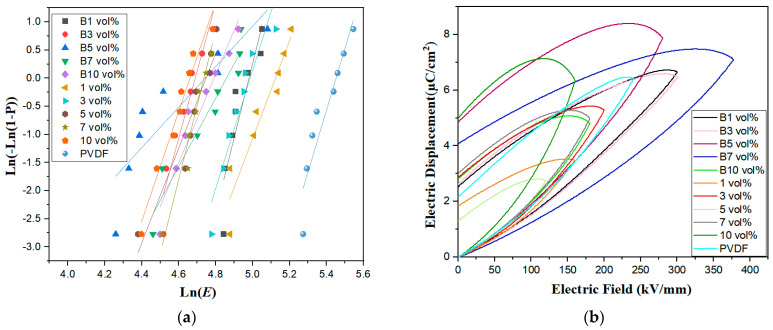
(**a**,**b**) Breakdown voltage and D–E hysteresis line of the composite film.

Figure 8a shows that a PVDF-based composite film with an increasing doping concentration corresponds to a decrease in the characteristic breakdown field strength, *E_b_*, value of the Weibull distribution due to the magnetic semiconductor high-entropy spinel ferrite (Mn_0.2_Zr_0.2_Cu_0.2_Ca_0.2_Ni_0.2_)Fe_2_O_4_ NFs’ filled phase, which is much more conductive than the PVDF-based polymer and, inevitably, forms conductive pathways, causing leakage currents to break through the film. It is also possible that as the doping concentration increases the crystalline region of PVDF changes, causing the PVDF molecular structure to change and introducing some defects detrimental to the breakdown strength of the composite media. The maximum breakdown strength of 1 vol% was 166 kV/mm, with a β of 10.26. B1 vol% had a maximum β-value of 13.47; additional data can be found in Table 2.

The capacitive energy storage properties of PVDF-based composite films have been investigated via the use of monopolar electric fields. Figure 8b shows the D–E hysteresis lines for PVDF-based composite films with (Mn_0.2_Zr_0.2_Cu_0.2_Ca_0.2_Ni_0.2_)Fe_2_O_4_ NF contents of 1 vol%, 3 vol%, 5 vol%, 7 vol%, and 10 vol%, without magnetic field treatment. As shown in Table 3, the maximum potential shifts under electric fields of 160 kV/mm, 200 kV/mm, 120 kV/mm, 180 kV/mm, and 160 kV/mm were 3.46 µC/cm^2^, 5.30 µC/cm^2^, 2.75 µC/cm^2^, 5.04 µC/cm^2^, and 6.32 µC/cm^2^, respectively. (Mn_0.2_Zr_0.2_Cu_0.2_Ca_0.2_Ni_0.2_)Fe_2_O_4_ NFs favored polarization, but the high residual polarization value, approximately 4.99 µC/cm^2^, combined with the large aggregation of the SEM nanofiber filler in the PVDF substrate, as shown in Figure 6e, and the α-phase of the XRD sample, shown in Figure 4, all contributed to (Mn_0.2_Zr_0.2_Cu_0.2_Ca_0.2_Ni_0.2_)Fe_2_O_4_ having a large residual polarization.

The maximum potential shifts in the composite films induced by the 1 vol%, 3 vol%, 5 vol%, 7 vol%, and 10 vol% magnetic fields at 300 kV/mm, 300 kV/mm, 280 kV/mm, 180 kV/mm, and 160 kV/mm electric fields were 6.65 µC/cm^2^, 6.47 µC/cm^2^, 7.9 µC/cm^2^, 7.11 µC/cm^2^, and 4.7 µC/cm^2^, respectively. They start with higher values for lower contents (1 vol% and 3 vol% compared with B1 and B3), as concentration increases the difference in Eb decreases (equal for the 5 vol%), and they end with higher values for B7 and B10 compared to 7 vol% and 10 vol% samples. The dielectric polarization of the magnetic-field-treated films was higher for doping concentrations less than or equal to 7 vol%, indicating that the magnetic field was favorable for dielectric polarization, with the greatest polarization occurring at 5 vol% of (Mn_0.2_Zr_0.2_Cu_0.2_Ca_0.2_Ni_0.2_)Fe_2_O_4_ NFs induced by the magnetic field. The residual polarization was about 4.84 µC/cm^2^. The magnetic-field-induced residual polarization of 1 vol% in the highly polarized samples was smaller, at about 2.52 µC/cm^2^, which yields a higher discharge energy density and charging/discharging efficiency.

**Figure 9:**
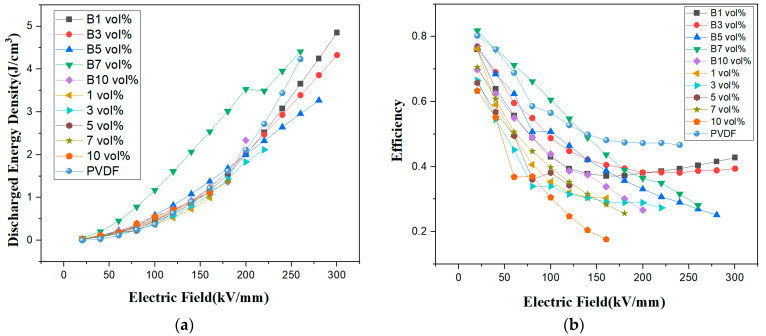
(**a**,**b**) Discharge energy density and efficiency of the composite film.

Figure 9a,b shows the discharge energy density and charge/discharge efficiency of the composite films. The top three composite films were magnetic-field-treated materials; in descending order of discharge energy density, they were 1 vol% (4.85 J/cm^3^), 7 vol% (4.41 J/cm^3^), and 3 vol% (4.33 J/cm^3^), all greater than pure PVDF (4.23 J/cm^3^). The magnetic-field-treated B1 vol% and B3 vol% composite films comingled the γ- and α-phases of PVDF, and the B5 vol% and B7 vol% composite films were γ-phase polymers. In the films without magnetic field treatment, the fillers were agglomerated in the PVDF substrate and prematurely saturated with polarization, resulting in a lower discharge energy density and efficiency. The magnetic field causes the agglomerated fibers to deconglomerate along the magnetic field direction and link into chains, which improve the interfacial compatibility with PVDF and increase the discharge energy density of the (Mn_0.2_Zr_0.2_Cu_0.2_Ca_0.2_Ni_0.2_)Fe_2_O_4_/PVDF nanocomposite film. A composite film with a high discharge energy density also has a high charge/discharge efficiency. The B1 vol% of the composite film has a charge/discharge efficiency of 43% at 300 kV/mm.

## 4. Conclusions

In this paper, (Mn_0.2_Zr_0.2_Cu_0.2_Ca_0.2_Ni_0.2_)Fe_2_O_4_ NFs were prepared via the sol–gel and electrostatic spinning methods, and (Mn_0.2_Zr_0.2_Cu_0.2_Ca_0.2_Ni_0.2_)Fe_2_O_4_/PVDF composite films were prepared via the use of the coating method. The effects of the magnetic field and high-entropy spinel ferrite (Mn_0.2_Zr_0.2_Cu_0.2_Ca_0.2_Ni_0.2_)Fe_2_O_4_ NF filler on the structural, dielectric, and energy storage properties of PVDF-based polymers were investigated. The experimental results show the strong influence of the magnetic field and (Mn_0.2_Zr_0.2_Cu_0.2_Ca_0.2_Ni_0.2_)Fe_2_O_4_ NFs on the phase of the polymer. The composite film contains α- and γ-, comingling, and γ-phase polymers. Structurally, the magnetic field treatment caused the originally agglomerated nanofibers in the PVDF polymer matrix to be linked end-to-end along the magnetic field direction to form a linear fiber chain, with different fiber chains running parallel to each other. Electrically, the application of the magnetic field enhanced the interfacial polarization and the (Mn_0.2_Zr_0.2_Cu_0.2_Ca_0.2_Ni_0.2_) Fe_2_O_4_/PVDF nanocomposite film. A doping concentration of B10 vol% had a maximum dielectric constant of 13.9, greater than the pure PVDF dielectric constant of 11. The impact on loss was very low; e.g., the low energy loss was 0.068. When the PVDF-based nanocomposite with (Mn_0.2_Zr_0.2_Cu_0.2_Ca_0.2_Ni_0.2_)Fe_2_O_4_ doping concentration was B1 vol%, the discharge energy density of the PVDF-based composite was improved to 4.85 J/cm^3^, which was greater than that of pure PVDF, at 4.23 J/cm^3^, with a charge/discharge efficiency of 43%.

## Figures and Tables

**Table 1:** XRF of (Mn_0.2_Zr_0.2_Cu_0.2_Ca_0.2_Ni_0.2_)Fe_2_O_4_ NFs.

Components	Mass %
Fe_2_O_3_	65.0138
MnO	5.9912
CaO	4.8054
NiO	5.7665
CuO	6.5011
ZrO_2_	11.3498

**Table 2:** *E_b_* and β of nanocomposite films.

Sample	*E_b_* (kV/mm)	β
0 vol%	226	12.49
1 vol%/B1 vol%	166/144	10.26/13.47
3 vol%/B3 vol%	145/108	9.58/11.87
5 vol%/B5 vol%	113/112	12.27/3.57
7 vol%/B7 vol%	113/126	16.54/7.67
10 vol%/B10 vol%	104/119	9.90/8.04

**Table 3:** Data of the D–E hysteresis loop of PVDF-based nanocomposite films.

Sample	Electric Fields (kV/mm)	Potential Shifts (µC/cm^2^)	Residual Polarization (µC/cm^2^)
0 vol%	260	6.38	2.15
1 vol%/B1 vol%	160/300	3.46/6.65	1.83/2.52
3 vol%/B3 vol%	200/300	5.30/6.47	2.86/2.62
5 vol%/B5 vol%	120/280	2.75/7.9	1.30/4.85
7 vol%/B7 vol%	180/180	5.04/7.11	3.01/4.07
10 vol%/B10 vol%	160/160	6.32/4.7	4.99/2.80

## Data Availability

Data are contained within the article. The data presented in this study are available in this paper.
